# Supplementary material for: Modelling vasopressin synthesis and storage dynamics during prolonged osmotic challenge and recovery based on activity dependent upregulation of mRNA transcription
Source: PLoS Comput Biol. 2026 Sep 30;22(9):e1014832. doi: 10.1371/journal.pcbi.1014832 (PMC13652331; doi:10.1371/journal.pcbi.1014832)
Supplement: Supplementary file 1 [file pcbi.1014832.s001.pdf]

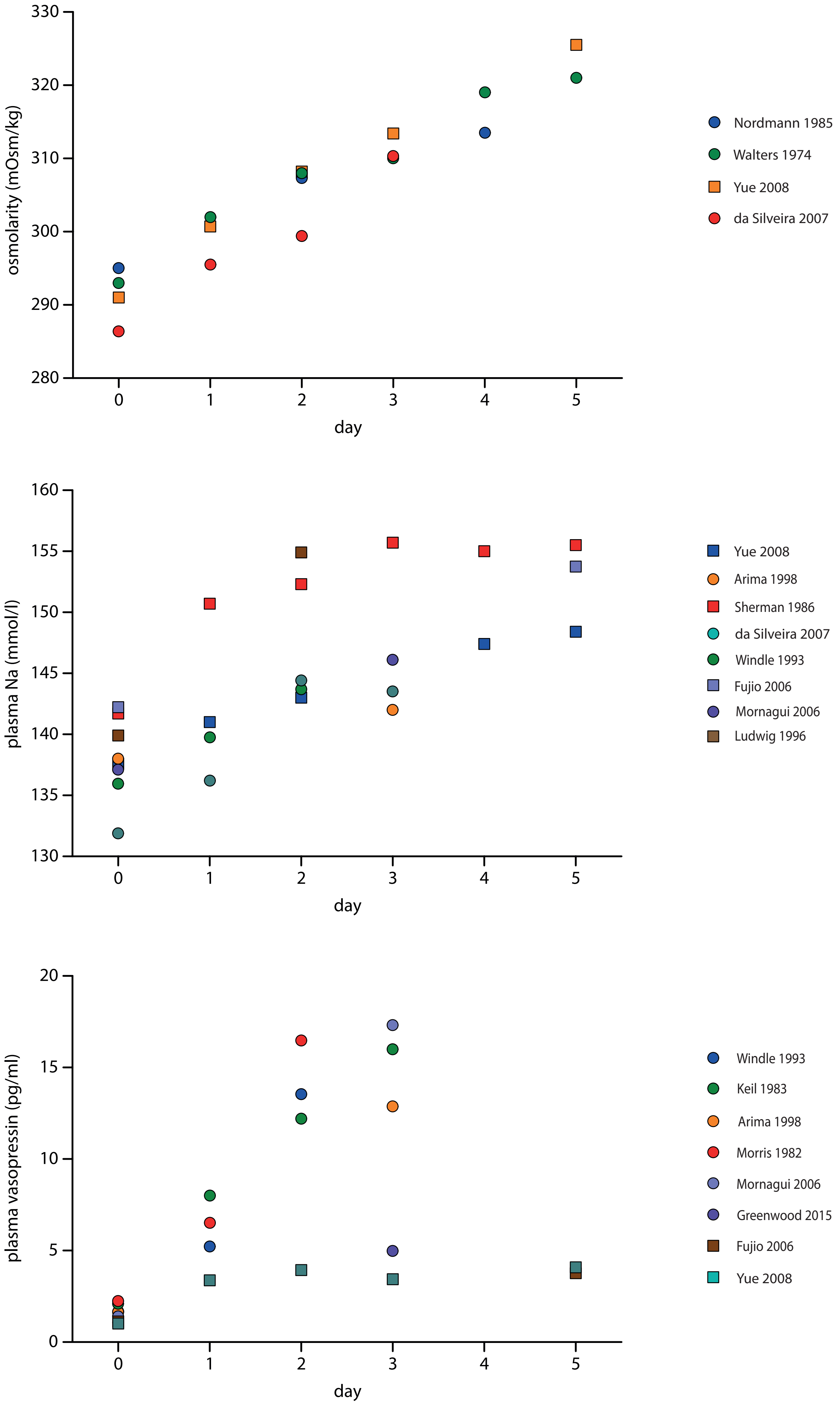

osmolarity (mOsm/kg)
330
320
310
300
290
280
0
1
2
3
4
5
day
Nordmann 1985
Walters 1974
Yue 2008
da Silveira 2007
plasma Na (mmol/l)
160
155
150
145
140
135
130
0
1
2
3
4
5
day
Yue 2008
Arima 1998
Sherman 1986
da Silveira 2007
Windle 1993
Fujio 2006
Mornagui 2006
Ludwig 1996
plasma vasopressin (pg/ml)
20
15
10
5
0
0
1
2
3
4
5
day
Windle 1993
Keil 1983
Arima 1998
Morris 1982
Mornagui 2006
Greenwood 2015
Fujio 2006
Yue 2008

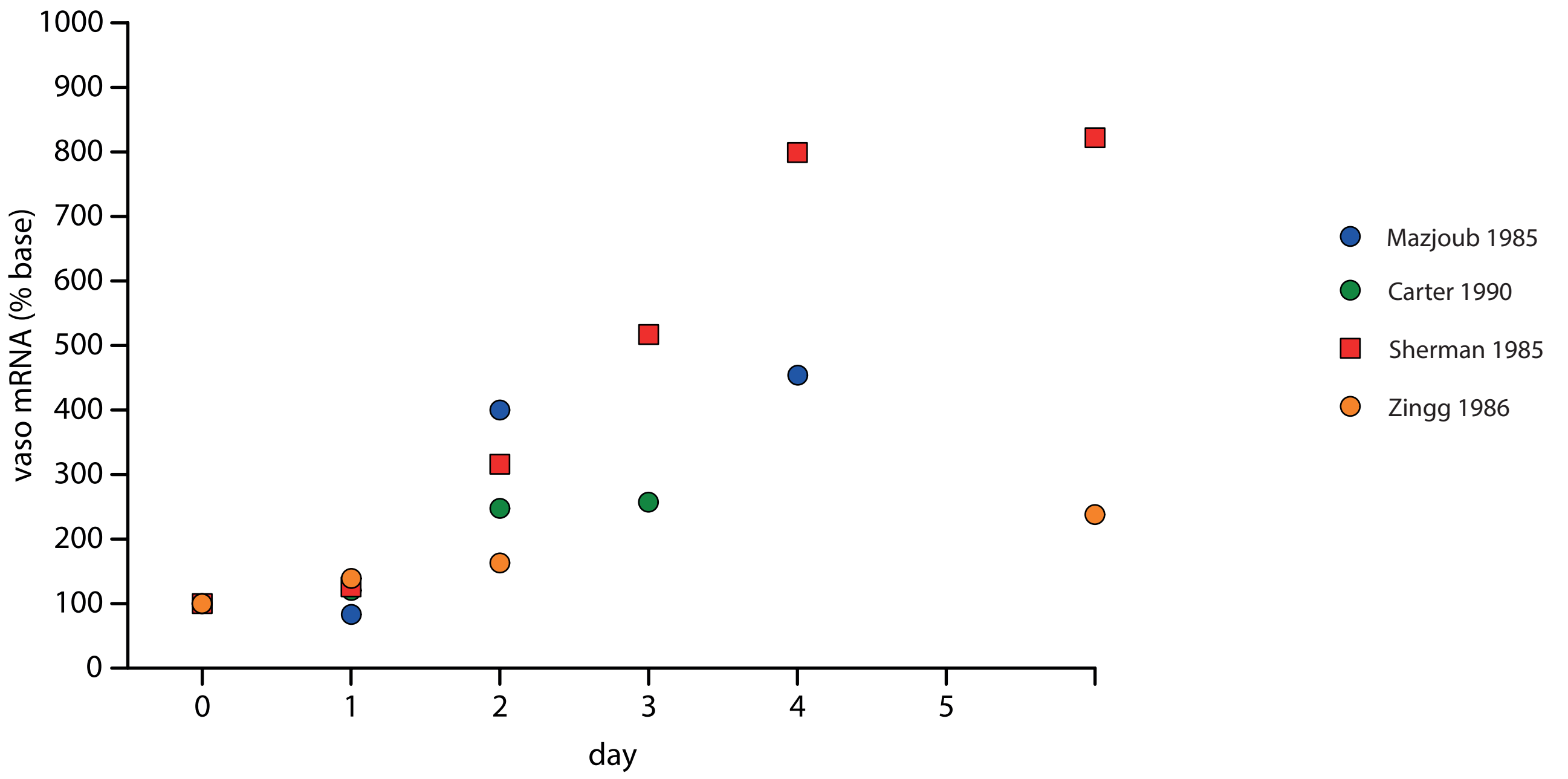

vaso mRNA (% base)
1000
900
800
700
600
500
400
300
200
100
0
0
1
2
3
4
5
day
Mazjoub 1985
Carter 1990
Sherman 1985
Zingg 1986

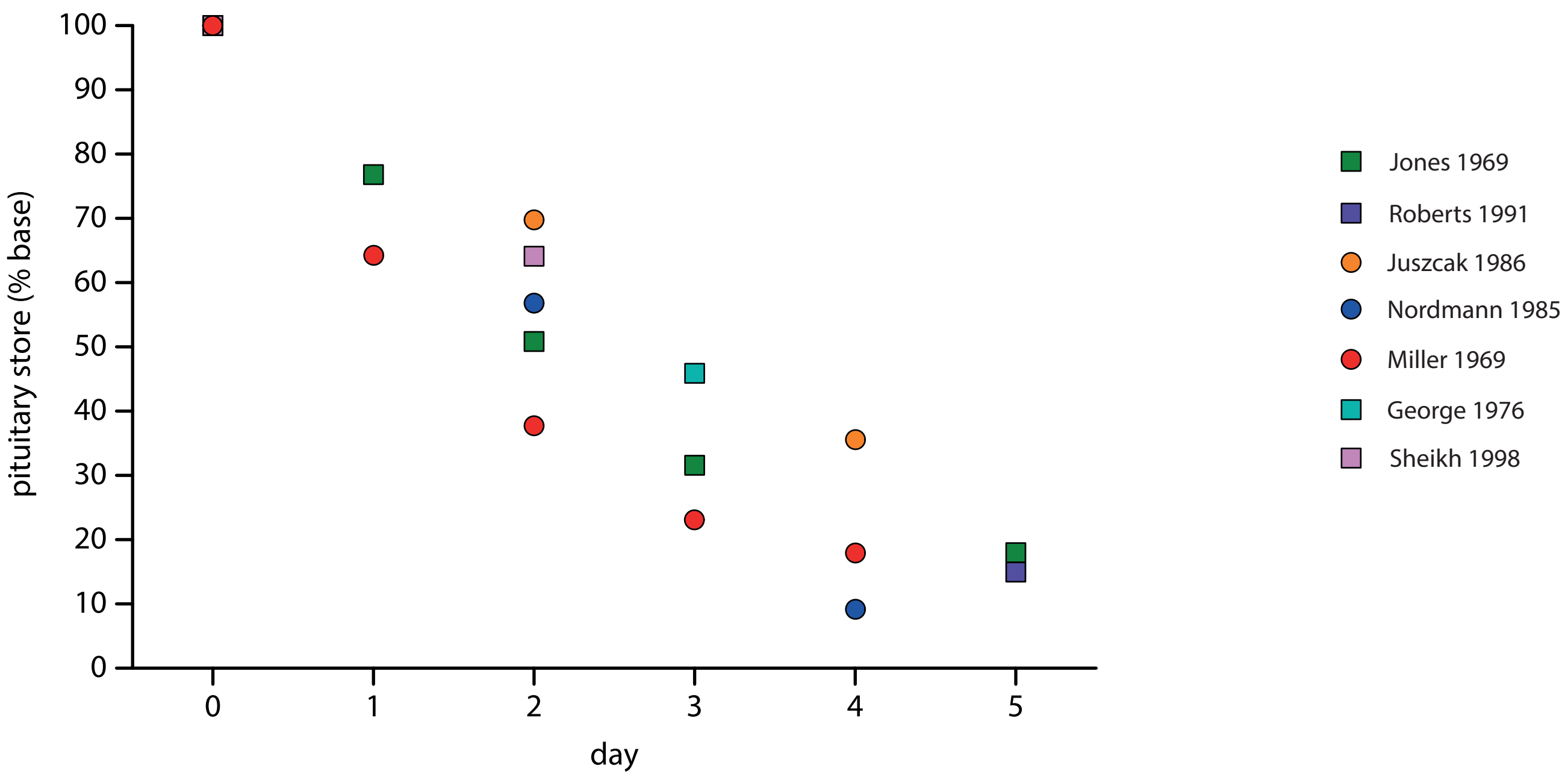

pituitary store (% base)
100
90
80
70
60
50
40
30
20
10
0
0
1
2
3
4
5
day
Jones 1969
Roberts 1991
Juszcak 1986
Nordmann 1985
Miller 1969
George 1976
Sheikh 1998

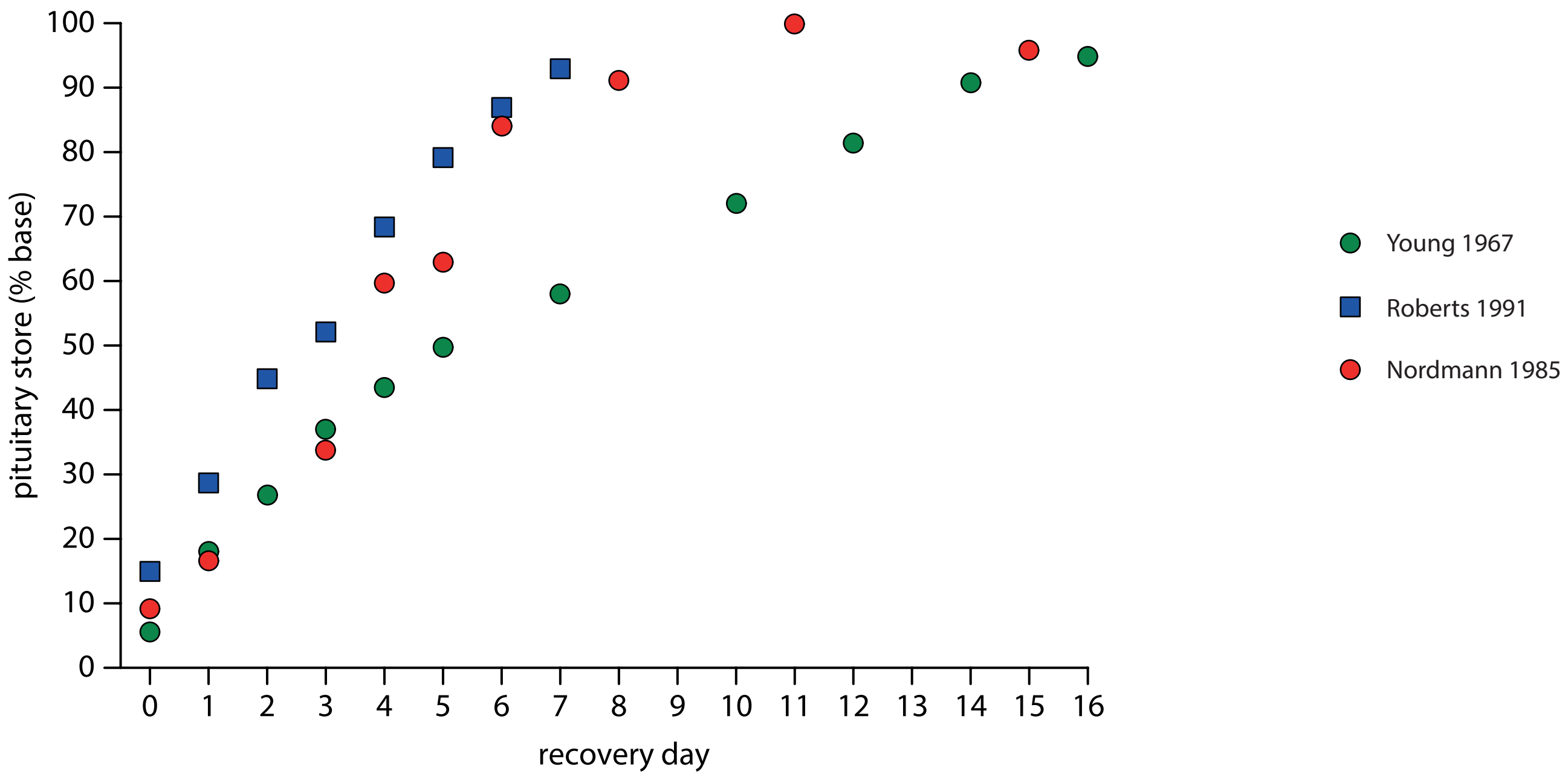

pituitary store (% base)
100
90
80
70
60
50
40
30
20
10
0
0 1 2 3 4 5 6 7 8 9 10 11 12 13 14 15 16
recovery day
Young 1967
Roberts 1991
Nordmann 1985
