# Supplementary material for: Modelling vasopressin synthesis and storage dynamics during prolonged osmotic challenge and recovery based on activity dependent upregulation of mRNA transcription
Source: PLoS Comput Biol. 2026 Sep 30;22(9):e1014832. doi: 10.1371/journal.pcbi.1014832 (PMC13652331; doi:10.1371/journal.pcbi.1014832)
Supplement: Supplementary file 2 [file pcbi.1014832.s004.doc]

**‘Modelling vasopressin synthesis and storage dynamics during prolonged osmotic challenge and recovery based on activity dependent upregulation of mRNA transcription’**

Duncan J. MacGregor, *Institute for Neuroscience and Cardiovascular Research, University of Edinburgh, George Square, Edinburgh, EH8 9XD, UK*

Dear Dr. Blackwell and Dr. Bush,

Thank you very much for considering my manuscript, and to the reviewers for the careful and encouraging comments. The detailed points of reviewer one have been useful towards improving the clarity of the figures and the methods, and reviewer two’s questions have been very good for further thinking and extending the discussion, with new sources. I believe I have made good use of and responded to all of the reviewers’ points. As requested, all the changes to the manuscript are highlighted and some figures have been revised to improve clarity of comparisons.

Details of Responses:

Reviewer #1

*1. Fig. 1: Need to say what the different colored symbols mean. Also, indicate in the lower panels when the challenge is in place and when there is recovery.*

The figure has been edited to add a legend explaining the square and circle symbols, and labels have been added to indicate challenge and recovery. Text has been added to the figure legend to explain the colours (sources detailed in Figure S1)

*2. Fig. 4: Some of the curves are very faint. Maybe increase the thickness or try using different colors. Also, in the caption, it should say that S_r=0 corresponds to the red curve.*

Apologies for this, I’d missed that some of the figure panels had not rendered correctly in the submitted PDF. This should be corrected with the submission of EPS versions of the figures. The figure legend has been corrected to associate red with s_r = 0.

*3. lines 314-316: It would help if you indicate the colors of the curves when discussing the different cases.*

Text has been added here to indicate colours as suggested.

*4. lines 409-410: I suppose you mean a comparison of vasopressin level in Fig. 5 (heterogeneous) versus that in Fig. 4 (homogeneous). To me, these look pretty similar, so I'm not sure that I agree that one is more nonlinear than the other.*

I’ve added overlaid data to figure 4 to show a homogeneous population alongside the single neuron. I’ve also added a homogeneous population with otherwise identical parameters (matching the adjusted input ramp) overlaid with the heterogeneous population data in figure 5. This should clarify the comparison between the homogeneous and heterogeneous plasma response and more clearly show the increase in linearity and dynamic range.

*5. Fig. 6: It would be helpful to explain why the red and blue curves in the bottom panel decline while the challenge is still present. Also, why does blue decline about an hour before red?*

Text has been added to the figure 6 legend to explain this effect, including the difference between blue and red. It’s essentially a drop off in response as some neurons’ stores become fully depleted, highlighting the eventual failure of a statically heterogeneous population, as described in the discussion.

*6. Methods: I don't see equations for vasopressin secretion.*

The equations are described in detail in our previous published work. I’ve added a clearer reference, but I’ve also added a new table putting all the current secretion and plasma model parameters, including adjustments described here, together.

*7. Methods: It would be good to show how Ire enters into the V differential equation. Overall, I don't really get how osmolarity is coded in terms of the input current. It would be good to give more details in Methods (the level of presentation in the text is fine).*

I’ve added text to the methods to clarify this, and better reference to the previous work where it is described in detail.

*8. There are no panel labels in any of the figures. I suppose this is okay, but panel labels are typical and they do make it easier to reference specific portions of the figures.*

I chose this style because many of the figures have multiple panels of the same type, and I think individual labels would do more harm in clutter than add clarity. Instead of letters there are more specific labels where I think them helpful.

Reviewer #2

*It has been reported that AVP synthesis (albeit measured as hypothalamic content), AVP storage, and AVP release from the pituitary exhibit daily rhythms in the rat (*[*https://doi.org/10.1677/joe.0.1330283*](https://eur02.safelinks.protection.outlook.com/?url=https%3A%2F%2Fdoi.org%2F10.1677%2Fjoe.0.1330283&data=05|02||b29a2f5dab5941e8981e08ded11162f5|2e9f06b016694589878910a06934dc61|0|0|639178069767339516|Unknown|TWFpbGZsb3d8eyJFbXB0eU1hcGkiOnRydWUsIlYiOiIwLjAuMDAwMCIsIlAiOiJXaW4zMiIsIkFOIjoiTWFpbCIsIldUIjoyfQ%3D%3D|0|||&sdata=EsniNrUbKSLkduxyjwLq311QCAlihLIWeGE%2F4I45Am8%3D&reserved=0)*). The data presented in this paper appear to fit well with your model. However, other studies have suggested that Avp synthesis in the SON and PVN does not exhibit a daily rhythm (*[*https://doi.org/10.1016/S0006-8993(00)03050-X*](https://eur02.safelinks.protection.outlook.com/?url=https%3A%2F%2Fdoi.org%2F10.1016%2FS0006-8993(00)03050-X&data=05|02||b29a2f5dab5941e8981e08ded11162f5|2e9f06b016694589878910a06934dc61|0|0|639178069767358586|Unknown|TWFpbGZsb3d8eyJFbXB0eU1hcGkiOnRydWUsIlYiOiIwLjAuMDAwMCIsIlAiOiJXaW4zMiIsIkFOIjoiTWFpbCIsIldUIjoyfQ%3D%3D|0|||&sdata=9VOhvWwLu3WgBsnkIBwF88W86%2BYQ5gOMeoXkYuj%2F31c%3D&reserved=0)*). Does your model suggest that the daily rhythm of Avp mRNA warrants further investigation? Additionally, is AVP neurone firing activity between the day and night phases different?*

I’ve added a paragraph to the discussion referencing these sources, and also work on circadian modulation of the osmotic input signal, suggesting future work to test this with the model. The mix of fast and slow dynamics in the model (particularly the mRNA pool) are such that these two sets of data are not necessarily inconsistent. There is also the caution that the Windle data is I think measuring the whole hypothalamus where it shows a daily rhythm in central vasopressin content.

*Have you considered incorporating peripheral feedback mechanisms into the model, given that receptors and ion channels are expressed on nerve terminals in the posterior pituitary? Could such mechanisms contribute to the modulation of AVP release, either promoting or attenuating secretion?*

This is done already in the secretion model which as well as including depletable stores, matches the non-linear coupling between spiking and secretion by including a combination of positive and negative feedback mechanisms. These explicitly include spike broadening, and Ca^2+^ dependent ion channel-based mechanisms. As discussed in the 2013 paper, other signals such as ATP were tested in the modelling, but didn’t change anything functionally, and so were left out in explicit form to keep the model simple.

*Do you think that neuronal memory or prior experience of a hyperosmotic challenge alters the response to subsequent osmotic challenges? Is there evidence to support this? For example, there is evidence that changes in AVP mRNA abundance during the rehydration phase following an osmotic challenge differ between the PVN and SON (doi:10.1371/journal.pone.0104802). Do you think the same principles incorporated in this model apply equally to AVP neurones in both the PVN and SON?*

Yes, and our recent electrophysiological study shows this, as well as the data referenced here. I’ve added a new discussion paragraph referencing this work and looking at this in the context of the current model. It is likely that the same form of mRNA dynamics apply to the SON and PVN, but with different parameters and degrees of heterogeneity.

Yours sincerely

Duncan MacGregor
