# Supplementary material for: Modelling vasopressin synthesis and storage dynamics during prolonged osmotic challenge and recovery based on activity dependent upregulation of mRNA transcription
Source: PLoS Comput Biol. 2026 Sep 30;22(9):e1014832. doi: 10.1371/journal.pcbi.1014832 (PMC13652331; doi:10.1371/journal.pcbi.1014832)
Supplement: Peer review history

# Peer review: Decision Letter 0

**DOI:** 10.1371/journal.pcbi.1014832.r001

**Authors:** Kim Blackwell, Daniel Bush

20 Jun 2026

PCOMPBIOL-D-26-01134

Modelling vasopressin synthesis and storage dynamics during prolonged osmotic challenge and recovery based on activity dependent upregulation of mRNA transcription

PLOS Computational Biology

Dear Dr. MacGregor,

Thank you for submitting your manuscript to PLOS Computational Biology. After careful consideration, we feel that it has merit but does not fully meet PLOS Computational Biology's publication criteria as it currently stands. Therefore, we invite you to submit a revised version of the manuscript that addresses the points raised during the review process.

Please submit your revised manuscript by Jul 20 2026 11:59PM. If you will need more time than this to complete your revisions, please reply to this message or contact the journal office at ploscompbiol@plos.org. When you're ready to submit your revision, log on to https://www.editorialmanager.com/pcompbiol/ and select the 'Submissions Needing Revision' folder to locate your manuscript file.

Please include the following items when submitting your revised manuscript:

* A letter that responds to each point raised by the editor and reviewer(s). You should upload this letter as a separate file labeled 'Response to Reviewers'. This file does not need to include responses to formatting updates and technical items listed in the 'Journal Requirements' section below.

* A marked-up copy of your manuscript that highlights changes made to the original version. You should upload this as a separate file labeled 'Revised Manuscript with Track Changes'.

* An unmarked version of your revised paper without tracked changes. You should upload this as a separate file labeled 'Manuscript'.

If you would like to make changes to your financial disclosure, competing interests statement, or data availability statement, please make these updates within the submission form at the time of resubmission. Guidelines for resubmitting your figure files are available below the reviewer comments at the end of this letter.

As the corresponding author, your ORCID iD is verified in the submission system and will appear in the published article. PLOS supports the use of ORCID, and we encourage all coauthors to register for an ORCID iD and use it as well. Please encourage your coauthors to verify their ORCID iD within the submission system before final acceptance, as unverified ORCID iDs will not appear in the published article. Only the individual author can complete the verification step; PLOS staff cannot verify ORCID iDs on behalf of authors.

We look forward to receiving your revised manuscript.

Kind regards,

Kim T. Blackwell, V.M.D., Ph.D.

Academic Editor

PLOS Computational Biology

Daniel Bush

Section Editor

PLOS Computational Biology

**Journal Requirements:**

If the reviewer comments include a recommendation to cite specific previously published works, please review and evaluate these publications to determine whether they are relevant and should be cited. There is no requirement to cite these works unless the editor has indicated otherwise.

1) We ask that a manuscript source file is provided at Revision. Please upload your manuscript file as a .doc, .docx, .rtf or .tex. If you are providing a .tex file, please upload it under the item type u2018LaTeX Source Fileu2019 and leave your .pdf version as the item type u2018Manuscriptu2019.

2) Please upload all main figures as separate Figure files in .tif or .eps format. For more information about how to convert and format your figure files please see our guidelines:

https://journals.plos.org/ploscompbiol/s/figures

3) Please upload a copy of Figure Fig 1-9 which you refer to in your text on pages 6, 9, 11, 12, 14, 17, 20, and 26. Or, if the figure is no longer to be included as part of the submission please remove all reference to it within the text.

4) We have noticed that you have uploaded Supporting Information files, but you have not included a list of legends. Please add a full list of legends for your Supporting Information files after the references list.

5) We have amended your Competing Interest statement to comply with journal style. We kindly ask that you double check the statement and let us know if anything is incorrect.

**Reviewers' comments:**

Reviewer's Responses to Questions

**Comments to the Authors:**

**Please note here if the review is uploaded as an attachment.**

Reviewer #1: This manuscript continues the development of a biophysically-based model of vasopressin hormone regulation that the author has pursued for more than a decade. He, along with his frequent collaborator Gareth Leng, is without a doubt the leader in the field in the modeling of vasopressin and oxytocin hormone regulation. This article extends the author's earlier model by adding a slow component to account for the depletion and recovery of vasopressin stores in the synapses. This is an important step since it is required for homeostasis of the system. These neurons release vasopressin in response to dehydration, which can last for several days. Somehow the cell must know when the vasopressin stores at the distant synapses begin to deplete so that synthesis can be increased; this model demonstrates how this can happen. It is well written, with a focus on the biology. It also clearly lays out assumptions and simplifications made in the model. I have several comments that I think will improve the presentation.

1. Fig. 1: Need to say what the different colored symbols mean. Also, indicate in the lower panels when the challenge is in place and when there is recovery.

2. Fig. 4: Some of the curves are very faint. Maybe increase the thickness or try using different colors. Also, in the caption, it should say that S_r=0 corresponds to the red curve.

3. lines 314-316: It would help if you indicate the colors of the curves when discussing the different cases.

4. lines 409-410: I suppose you mean a comparison of vasopressin level in Fig. 5 (heterogeneous) versus that in Fig. 4 (homogeneous). To me, these look pretty similar, so I'm not sure that I agree that one is more nonlinear than the other.

5. Fig. 6: It would be helpful to explain why the red and blue curves in the bottom panel decline while the challenge is still present. Also, why does blue decline about an hour before red?

6. Methods: I don't see equations for vasopressin secretion.

7. Methods: It would be good to show how Ire enters into the V differential equation. Overall, I don't really get how osmolarity is coded in terms of the input current. It would be good to give more details in Methods (the level of presentation in the text is fine).

8. There are no panel labels in any of the figures. I suppose this is okay, but panel labels are typical and they do make it easier to reference specific portions of the figures.

Reviewer #2: This manuscript addresses a highly pertinent and interesting question. The model effectively incorporates AVP synthesis by magnocellular neurones, AVP storage in the posterior lobe of the pituitary gland, and AVP release into the peripheral circulation. This work adds substantial value to the field, as most studies focus on individual components of this complex axis rather than integrating them into a single framework. The computational model presented here facilitates improved interpretation of both previously published data and future findings. The author has developed an excellent model system with which to interrogate these processes.

I have a couple of points for the author.

It has been reported that AVP synthesis (albeit measured as hypothalamic content), AVP storage, and AVP release from the pituitary exhibit daily rhythms in the rat (https://doi.org/10.1677/joe.0.1330283). The data presented in this paper appear to fit well with your model. However, other studies have suggested that Avp synthesis in the SON and PVN does not exhibit a daily rhythm (https://doi.org/10.1016/S0006-8993(00)03050-X). Does your model suggest that the daily rhythm of Avp mRNA warrants further investigation? Additionally, is AVP neurone firing activity between the day and night phases different?

Have you considered incorporating peripheral feedback mechanisms into the model, given that receptors and ion channels are expressed on nerve terminals in the posterior pituitary? Could such mechanisms contribute to the modulation of AVP release, either promoting or attenuating secretion?

Do you think that neuronal memory or prior experience of a hyperosmotic challenge alters the response to subsequent osmotic challenges? Is there evidence to support this? For example, there is evidence that changes in AVP mRNA abundance during the rehydration phase following an osmotic challenge differ between the PVN and SON (doi:10.1371/journal.pone.0104802). Do you think the same principles incorporated in this model apply equally to AVP neurones in both the PVN and SON?

**********

**Have the authors made all data and (if applicable) computational code underlying the findings in their manuscript fully available?**

The PLOS Data policy requires authors to make all data and code underlying the findings described in their manuscript fully available without restriction, with rare exception (please refer to the Data Availability Statement in the manuscript PDF file). The data and code should be provided as part of the manuscript or its supporting information, or deposited to a public repository. For example, in addition to summary statistics, the data points behind means, medians and variance measures should be available. If there are restrictions on publicly sharing data or code —e.g. participant privacy or use of data from a third party—those must be specified.

Reviewer #1: Yes

Reviewer #2: Yes

**********

PLOS authors have the option to publish the peer review history of their article (what does this mean?). If published, this will include your full peer review and any attached files.

If you choose “no”, your identity will remain anonymous but your review may still be made public.

**Do you want your identity to be public for this peer review?**  For information about this choice, including consent withdrawal, please see our Privacy Policy.

Reviewer #1: No

Reviewer #2: No

[NOTE: If reviewer comments were submitted as an attachment file, they will be attached to this email and accessible via the submission site. Please log into your account, locate the manuscript record, and check for the action link "View Attachments". If this link does not appear, there are no attachment files.]

**Figure resubmission:**

**Reproducibility:**

To enhance the reproducibility of your results, we recommend that authors of applicable studies deposit laboratory protocols in protocols.io, where a protocol can be assigned its own identifier (DOI) such that it can be cited independently in the future. Additionally, PLOS ONE offers an option to publish peer-reviewed clinical study protocols. Read more information on sharing protocols at https://plos.org/protocols?utm_medium=editorial-email&utm_source=authorletters&utm_campaign=protocols

# Author comment: Author response to Decision Letter 1

**DOI:** 10.1371/journal.pcbi.1014832.r002

24 Aug 2026

Attachment Submitted filename: macgregor synthesis response letter.doc

# Peer review: Decision Letter 1

**DOI:** 10.1371/journal.pcbi.1014832.r003

**Authors:** Kim Blackwell, Daniel Bush

16 Sep 2026

Dear Dr MacGregor,

We are pleased to inform you that your manuscript 'Modelling vasopressin synthesis and storage dynamics during prolonged osmotic challenge and recovery based on activity dependent upregulation of mRNA transcription' has been provisionally accepted for publication in PLOS Computational Biology.

Before your manuscript can be formally accepted you will need to complete some formatting changes, which you will receive in a follow up email. A member of our team will be in touch with a set of requests.

Please note that your manuscript will not be scheduled for publication until you have made the required changes, so a swift response is appreciated.

IMPORTANT: The editorial review process is now complete. PLOS will only permit corrections to spelling, formatting or significant scientific errors from this point onwards. Requests for major changes, or any which affect the scientific understanding of your work, will cause delays to the publication date of your manuscript.

Should you, your institution's press office or the journal office choose to press release your paper, you will automatically be opted out of early publication. We ask that you notify us now if you or your institution is planning to press release the article. All press must be co-ordinated with PLOS.

Thank you again for supporting Open Access publishing; we are looking forward to publishing your work in PLOS Computational Biology.

Best regards,

Kim T. Blackwell, V.M.D., Ph.D.

Academic Editor

PLOS Computational Biology

Daniel Bush

Section Editor

PLOS Computational Biology

***********************************************************

Reviewer's Responses to Questions

**Comments to the Authors:**

**Please note here if the review is uploaded as an attachment.**

Reviewer #1: Nicely done.

Reviewer #2: The author has successfully addressed my comments.

**********

**Have the authors made all data and (if applicable) computational code underlying the findings in their manuscript fully available?**

The PLOS Data policy requires authors to make all data and code underlying the findings described in their manuscript fully available without restriction, with rare exception (please refer to the Data Availability Statement in the manuscript PDF file). The data and code should be provided as part of the manuscript or its supporting information, or deposited to a public repository. For example, in addition to summary statistics, the data points behind means, medians and variance measures should be available. If there are restrictions on publicly sharing data or code —e.g. participant privacy or use of data from a third party—those must be specified.

Reviewer #1: None

Reviewer #2: Yes

**********

PLOS authors have the option to publish the peer review history of their article (what does this mean?). If published, this will include your full peer review and any attached files.

If you choose “no”, your identity will remain anonymous but your review may still be made public.

**Do you want your identity to be public for this peer review?**  For information about this choice, including consent withdrawal, please see our Privacy Policy.

Reviewer #1: No

Reviewer #2: No

# Editor report: Acceptance letter

**DOI:** 10.1371/journal.pcbi.1014832.r004

**Authors:** Kim Blackwell, Daniel Bush

PCOMPBIOL-D-26-01134R1

Modelling vasopressin synthesis and storage dynamics during prolonged osmotic challenge and recovery based on activity dependent upregulation of mRNA transcription

Dear Dr MacGregor,

I am pleased to inform you that your manuscript has been formally accepted for publication in PLOS Computational Biology. Your manuscript is now with our production department and you will be notified of the publication date in due course.

The corresponding author will soon be receiving a typeset proof for review, to ensure errors have not been introduced during production. Please review the PDF proof of your manuscript carefully, as this is the last chance to correct any errors. Please note that major changes, or those which affect the scientific understanding of the work, will likely cause delays to the publication date of your manuscript.

Soon after your final files are uploaded, unless you have opted out, the early version of your manuscript will be published online. The date of the early version will be your article's publication date. The final article will be published to the same URL, and all versions of the paper will be accessible to readers.

For Research, Software, and Methods articles, you will receive an invoice from PLOS for your publication fee after your manuscript has reached the completed accept phase. If you receive an email requesting payment before acceptance or for any other service, this may be a phishing scheme. Learn how to identify phishing emails and protect your accounts at https://explore.plos.org/phishing.

Thank you again for supporting PLOS Computational Biology and open-access publishing. We are looking forward to publishing your work!

With kind regards,

Sharmila Kamatchi

PLOS Computational Biology | Carlyle House, Carlyle Road, Cambridge CB4 3DN | United Kingdom ploscompbiol@plos.org | Phone +44 (0) 1223-442824 | ploscompbiol.org | @PLOSCompBiol
